# From Prognostic Marker to Therapeutic Agent: The Role of Nitric Oxide in Lung Cancer

**DOI:** 10.3390/jcm14196801

**Published:** 2025-09-26

**Authors:** Tommaso Pianigiani, Akter Dilroba, Asia Fanella, Laura Bergantini, Miriana d’Alessandro, Elena Bargagli, Paolo Cameli

**Affiliations:** Respiratory Diseases Unit, Department of Medicine, Surgery and Neurosciences, University of Siena, 53100 Siena, Italy; tommaso.pianigian@student.unisi.it (T.P.); a.dilroba@student.unisi.it (A.D.); a.fanella@student.unisi.it (A.F.); laura.bergantini@unisi.it (L.B.); miriana.dalessandro@unisi.it (M.d.); bargagli2@unisi.it (E.B.)

**Keywords:** nitric oxide, lung cancer, immunooncology, biomarker, novel therapeutic approaches, fractional exhaled nitric oxide

## Abstract

**Background**: Nitric oxide (NO) is a gaseous free radical produced from L-arginine by the nitric oxide synthase (NOS) enzymes. NO exerts a dose-dependent biphasic effect on lung cancer development, angiogenesis, and dissemination. The widespread contribution of nitric oxide signaling to lung cancer biology has cast a spotlight on the identification of NO-based therapeutic approaches as well as the use of fractional exhaled NO (FeNO) as a prognostic biomarker of clinical control. However, the significance of lung cancer treatment and prognosis has not been fully elucidated. **Objective**: This narrative review gives an overview of NO in lung cancer, focusing on its therapeutic and prognostic implications. **Results**: FeNO may help to assess the complications associated with non-pharmacological treatments, including postoperative pneumonia and radiation pneumonitis. By contrast, the role of FeNO dynamics during pharmacological treatment is still largely unexplored due to the suppressive effect of chemotherapy on FeNO levels. The rise of immunotherapy may pave the way to a better evaluation of FeNO as a prognostic biomarker of treatment response. The dichotomous involvement of NO in lung cancer events has led to the adoption of several NO-centered treatments that are focused on both inhibiting and enhancing NO signaling. However, NO chemical and biological characteristics have hindered its implementation in clinical practice. **Conclusions**: In the coming years, the advancements in drug delivery systems may lead to more effective anti-cancer applications of NO by improving tumor targeting and minimizing the systemic side effects. Together, our findings emphasize the promising role of NO in lung cancer treatment, underscoring the challenges and avenues for future research.

## 1. Introduction

### 1.1. Nitric Oxide Signaling and Nitrosative Stress in Respiratory Pathophysiology

Nitric oxide (NO) is a gaseous free radical molecule that is produced from L-arginine by the action of various NADPH-dependent enzymes called NO synthases (NOSs) [1]. Unlike other isoforms, inducible NOS (iNOS) is not usually expressed in cells, but its expression can be induced by different triggers, such as immunostimulatory cytokines or bacterial products, in a wide variety of cell types [2] (Table 1).

Once iNOS is expressed, it is constitutively activated, producing unregulated high concentrations of NO in a short time frame [2]. The overproduction of NO by iNOS can either produce positive or negative biological effects, dependent upon its concentration and the organs involved. The main signaling pathway carried out by NO is through the S-nitrosylation of proteins, regulating several cellular mechanisms, including membrane trafficking and protein phosphorylation, and, when dysregulated, plays a key role in a broad spectrum of human diseases [3,4]. Moreover, increased NOS activity leads to the excess formation of reactive nitrogen species, like peroxynitrite, resulting in cellular nitrosative stress [5,6]. Nitrosative stress promotes protein tyrosine nitration, eliciting many detrimental effects on cellular homeostasis, such as the activation of cascade signal responses of cell death and DNA strand breaks [7]. Over the last decades, the biochemical mechanisms underlying NO signaling dysregulation and nitrosative stress have attracted considerable attention [8,9]. Scientific inquiries have embarked on the development of NO-based novel therapeutic approaches, including inhibitors of endogenous NO synthesis, scavengers of NO, and prodrugs elevating NO levels [10]. NO is also a key regulator of respiratory tract homeostasis, which is involved in a countless number of physiological and pathophysiological processes, including antimicrobial protection, blood flow regulation, modulation of bronchial reactivity, and mucociliary activity [11,12]. Dysregulated NO signaling may result in noxious effects such as airway inflammation, bronchial hyperreactivity, and mucus hypersecretion [13,14], thus correlating NO with the pathogenesis of many respiratory diseases [1,14,15,16,17,18]. Fractional exhaled NO (FeNO) is a non-invasive test that measures the amount of NO produced in the airways and is currently recommended in the diagnostic work-up and therapeutic management of bronchial asthma [19,20]. The enhanced comprehension of different disease pathways may lead to an expansion of their clinical use to a broader spectrum of respiratory diseases, including interstitial lung diseases [21] and chronic obstructive pulmonary disease [22], with promising results.

### 1.2. Nitric Oxide Signaling in Lung Cancer: Mechanisms, Therapeutic Potential, and Challenges

**Figure 1:**
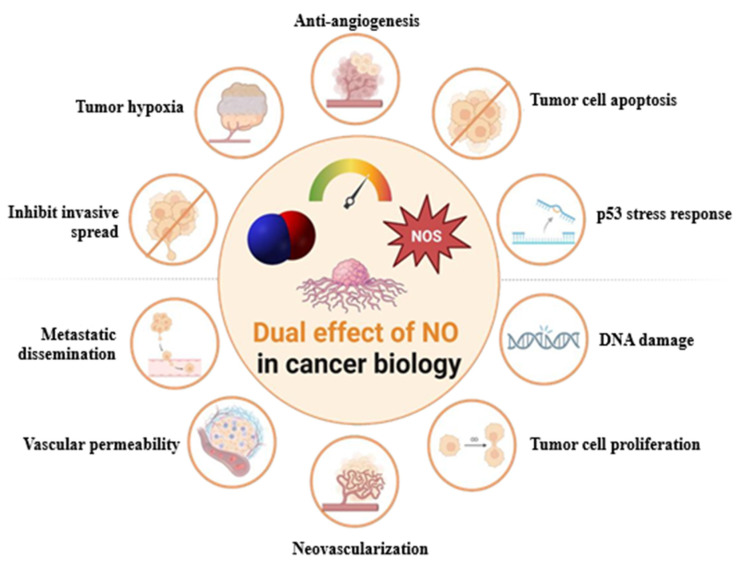
Dose-dependent biphasic effect of NO on cancer development, cell proliferation, angiogenesis, and dissemination.

The pleiotropic activity of NO reflects its multifaceted role in tumor biology [23]. The complex network of interactions between NO and the tumor microenvironment contributes to cancer proliferation, angiogenesis, metastasis, and anti-tumor immunity [24,25]. Depending on several factors, including the duration and cellular sensitivity to exposure, NO can exert both tumoricidal and tumor-promoting responses [23,25] (Figure 1).

Lung cancer is the leading cause of cancer-related deaths worldwide. Although lung cancer pathogenesis is considered a multiphase phenomenon depending on the interplay of genetics with environmental factors [26], it has been widely accepted that NO plays a master role in different lung cancer-related events [27]. Indeed, cigarette smoking, a major cause of lung cancer, associated with more than 85 percent of lung cancers [26], induced a huge exposure to reactive oxygen/nitrogen species, boosting oxidative/nitrosative stress to the bronchial and alveolar tissues [28]. Moreover, prolonged exposure to cigarette smoking may lead to the upregulation of iNOS, further enhancing pulmonary NO production [29].

During the last decades, several studies have explored the contribution of NO accumulation to human lung carcinoma, reporting discordant results.

Given the dualism of NO activity based on its concentration, a better understanding of the molecular mechanisms underlying NO activity on cancer cells and the tumor environment could potentially lead to the development of novel therapeutic agents for the treatment of lung cancer [24]. Low and intermediate concentrations of NO promote cancer cell progression, prevent apoptosis, stimulate angiogenesis and metastasis, while higher concentrations of NO have an anti-tumor effect by enhancing apoptosis and the efficacy of chemotherapy and radiotherapy on cancer cells [25]. This leads to the idea of developing targeted therapy based on NO [1]: exogenous delivery of NO can add up to endogenous NOS-derived sources, raising the concentrations of NO to cytotoxic levels against neoplastic cells, whereas inhibiting NO or its production can block tumor-promoting activity.

Studies based on the use of NO have been carried out using in vitro and in vivo models for the treatment of various tumors, such as prostate cancer, breast cancer, and neuroblastoma [10,29]. The main approach was focused on modulating the amount of NO exposed to cells and included viral transfection of genes, cell-based methods, NO prodrug, free radical scavengers, and pharmacological inhibition of NOS and NO itself [1].

However, even though the widespread contribution of NO to cancer-related events supports the potential adoption of NO-centered treatments [30], NO’s chemical and biological characteristics hinder their implementation in clinical practice [29]. NO has a very short half-life under physiological conditions and participates in different physiological processes of the body by modulating biochemical reactions, such as neurotransmission, blood flow regulation, inflammation, pain, immune responses, and gastroprotection. Given the assumptions, controlled delivery of NO-centered therapies is mandatory to limit systemic side effects and maximize anti-cancer effectiveness [23].

In recent decades, the potential of NO-based anti-cancer treatments has expanded with the development of more stable and optimized NO-based therapies [31]. In parallel, recent advancements in cancer therapies pose a significant challenge due to the lack of biomarkers capable of reliably predicting the course or progression of the disease.

The broad involvement of NO signaling in lung cancer biology raised the scientific interest of FeNO testing as a potentially simple and quantitative method to better assess anti-cancer treatment responses, as well as to intercept chemotoxicity and/or radiation toxicity early [32]. However, the prognostic implications of FeNO dynamics still raise controversy about the usefulness of this marker, mainly due to the heterogeneity of the available studies and the factors influencing its concentration [32,33].

The aim of this review is to provide an overview of the therapeutic and prognostic effectiveness of NO-based clinical strategies, encompassing the insights, limitations, challenges, and avenues for future research.

## 2. FeNO as a Biomarker of Treatment Response

In recent years, FeNO has been investigated in several studies that sought to determine whether it could be used as a prognostic marker for treatment response in cancer patients [32]. Its implementation in clinical practice was investigated in three main areas of cancer treatment: surgery, radiotherapy (RT), and pharmacological treatment.

### 2.1. Surgery

**Figure 2:**
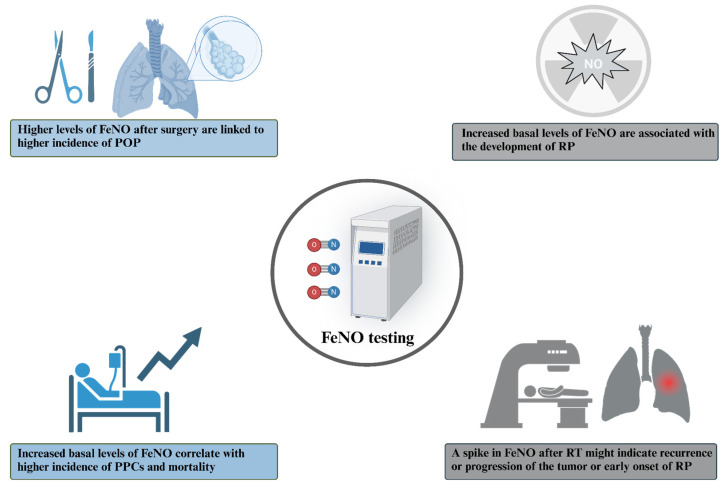
Potential implementation of FeNO testing in the assessment of complications associated with surgical treatment and RT.

Since airway inflammation could be linked to an increase in postoperative complications, FeNO could possibly be used as a prognostic marker in patients undergoing lung resection. A study conducted by Okamoto et al. discovered that the postoperative levels of FeNO were increased in all patients, regardless of the type of surgery performed, but the patients with higher levels of FeNO before surgery had an increased incidence of potentially preventable complications, which, in turn, correlated with mortality [34]. Similarly, a study by Liu et al. found that FeNO significantly increased in the patients who developed postoperative pneumonia (POP) [35]. Lastly, Lin et al. demonstrated that elevated preoperative levels of FeNO are independent risk factors for the development of a postoperative cough. Patients with high preoperative FeNO levels exhibited a significantly poorer cough-related quality of life and experienced delayed resolution of their postoperative cough symptoms [36] (Figure 2).

### 2.2. Radiotherapy

The opportunity of using FeNO as a prognostic marker for lung inflammation also led to the development of studies that focused on RT and its complications. Pulmonary toxicity is a well-known side effect of RT, which limits the dose that can be administered to patients and can impact their quality of life [37]. One of the manifestations of lung toxicity is radiation pneumonitis (RP), which is an inflammatory reaction that occurs within irradiated lung tissue [38]. The symptoms include cough, fever, shortness of breath, and changes in pulmonary function, and in rare cases, may be fatal. Several studies underlined the necessity to identify additional biomarkers to allow an early detection of lung toxicity and RP [39]. Some studies, like the one carried out by McCurdy et al., found that eNO could represent a valuable, rapid and cost-effective tool that was able to predict symptomatic RP with an anticipation of weeks to months before peak symptoms [40,41], whereas Enache et al. found that, eight months after the RT session, a spike in FeNO might indicate recurrence or progression of lung cancer as well as lung injury (such as RP) [42]. On the contrary, Szejniuk et al. found no difference in the FeNO levels between patients who developed RP and those who did not after high-dose RT, but still, patients who developed RP showed higher basal levels of FeNO that persisted throughout the RT and follow-up period [43] (Figure 2).

### 2.3. Chemotherapy

Chemotherapy (CHT) has also been associated with a reduction in FeNO after a couple of days from its administration [44], likely due to the reduction in monocytes in peripheral blood, which, in turn, is caused by the cell death in the tumor and the change in the microenvironment [45]. Certain CHT drugs, such as cisplatin, induce the production of reactive oxygen species (ROS); therefore, in patients undergoing CHT, the amount of ROS produced might overpower the antioxidant systems, thus resulting in diminished anti-tumoral activity [46]. Srivasta et al. reported that NO increased after CHT, whereas the antioxidant system concentration decreased proportionally to the severity of the cancer [47]. This finding was also described by Wevel et al., who sought to understand whether CHT caused a fall in FeNO and if that decrease might be caused by a reduction in monocytes in peripheral blood. Interestingly, the levels of FeNO were reduced concurrently with the fall of monocytes in peripheral blood, though not in a statistically significant manner [48].

To our knowledge, there are no available studies that correlate FeNO as a prognostic marker of progression in patients undergoing CHT. This might be an interesting field to explore in the future because of the low invasiveness and reliability of FeNO in measuring airway inflammation.

### 2.4. Immune Checkpoint-Inhibitors

Some studies have investigated the potential of FeNO or multiple-flows eNO parameters in assessing or predicting respiratory complications secondary to immune checkpoint inhibitor (ICI) treatment. A case report described a reliable and timely increase in FeNO in patients experiencing checkpoint inhibitor pneumonitis (CIP), suggesting its implementation in clinical practice for the early detection of this potentially dreadful complication [49]. Interestingly, FeNO appears to retain its well-established association with type 2 inflammation, as it was reported to be increased also in ICIs-treated patients developing chronic eosinophilic pneumonia, asthma exacerbation, or eosinophilic airway inflammation [50,51,52]. Supporting these preliminary findings, a very recent paper by Gao and colleagues proposed a CIP diagnostic model based on many parameters, among them CaNO. Notably, CaNO values >6.35 ppb were independently associated with CIP [53]; and even if promising, these findings will need to be further validated in prospective multicentric cohorts.

No data are currently available concerning the potential role of FeNO in predicting a good response to ICIs: in this regard, a pilot study, whose main outcome is to address FeNO as a prognostic marker of response to anti-PD-L1 immunotherapy in NSCLC, is actually recruiting (NCT05985330).

### 2.5. Limitations and Confounding Factors

In recent years, with the rise of personalized medicine, FeNO’s role has evolved significantly, no longer limited to serving as a static diagnostic or prognostic marker, but rather as a dynamic biomarker to guide individualized therapy. Elevated FeNO levels are strongly associated with T2-high inflammation, thus routinely enabling clinicians to make precise decisions regarding the use of inhaled corticosteroids (ICS) or biologic therapies, with superior outcomes over an approach that is based solely on asthma symptoms [54,55].

Overall, when it comes to using FeNO as a marker in cancer patients, we must also take into account some of its limitations, such as the fact that patients with asthma, eosinophilic airway inflammation, or high levels of allergen exposure, as well as patients with exacerbated chronic obstructive pulmonary disease, are known to express elevated FeNO levels, whereas inhaled and oral corticosteroids and cigarette smoke cause the opposite effect [56].

It must also be noted that the studies mentioned above attempted to find an FeNO cut-off level that could predict complications with acceptable sensitivity; however, it was not possible to reach a unified number. Subsequent studies with larger cohorts might be helpful in shedding light on this topic and will help define a “subgroup of high-risk patients” using an inexpensive and easily accessible tool such as FeNO.

Future studies might also shed light on the use of measuring FeNO at multiple flow rates—a tool that enables a partitioning of the measure of FeNO into its bronchial and alveolar sources. Lower flow rates reflect bronchial FeNO production (J’awNO) and are, therefore, associated with the bronchial compartment, which is more useful in asthma management [57,58].

CaNO is instead linked to the alveolar compartment and might therefore be useful in detecting parenchymal inflammation or tissue damage caused by systemic diseases affecting the lung [21,59]. These extended measurements appear to be a promising tool that might help explain more accurately the cause of FeNO elevation in every single patient. Their use is, however, not standardized at present and, thus, requires further validation in large clinical trials.

## 3. NO-Centered Therapeutic Approaches in Lung Cancer

### 3.1. Anti-NO Therapeutic Strategies

Given that low levels of NO promote tumor cell proliferation, migration, and angiogenesis, one strategy to prevent tumorigenesis is to inhibit NO production by targeting the molecular pathways involved. Indeed, iNOS is more expressed in lung tumors than in surrounding normal tissues, and its expression correlates with angiogenic status and metastatic range in tumors [60], suggesting NOS enzymes as potential new therapeutic targets in lung cancer.

Silibinin, a major constituent in silymarin, has a chemopreventive and antiangiogenic efficacy on urethane-induced lung tumorigenesis in terms of tumor growth and progression [61]. Indeed, studies on mouse models supported iNOS as a potential chemopreventive target during lung carcinogenesis as sibilinin decreases lung tumor multiplicity by 71% in wild-type mice but failed to have such efficacy in iNOS^−/−^ mice. iNOS is a target of silibinin-mediated action, and the antiangiogenetic role of silibinin is related to iNOS-dependent suppression of VEGF and its receptor, which are highly expressed in lung tumors [62,63]. In addition, the iNOS gene promoter region has binding sites for STAT, NF-κB, and HIF-1α, and silibinin inhibition of these transcription factors leads to the downregulation of iNOS expression observed in lung cancer cells [62].

In terms of inhibition of NO production, NG-nitro-L-arginine methyl ester (L-NAME) is one of the most clinically developed pan-NOS inhibitors for the treatment of septic and cardiogenic shock, evaluated for the treatment of lung cancer in a genetically engineered mouse model of KRAS mutation-positive non-small cell lung cancer (NSCLC). The results showed that L-NAME treatment inhibits lung growth, reduces tumor burden, and increases the median overall survival, especially if administered after platinum-based chemotherapeutics [64]. A recent study demonstrated that the combination of L-NAME and Hypericum alpestre extracts exerts an inhibitory effect on the PI3K/Akt signaling pathway, thereby enhancing apoptotic activity and exhibiting anti-cancer potential in A549 lung adenocarcinoma cells [65].

On the other hand, β-Elemene (the active component of elemene, extracted from the Chinese medicinal herb Curcuma Wenyujin) used in conjunction with radiotherapy, is an effective tool to overcome radioresistance in NSCLC [66]. β-Elemene reduces the expression of EMT/CSC markers and inhibits the Prx-1/NF-kB/iNOS signaling pathway. Β-Elemene blocks the translocation of NF-κB p50/p65 from cytoplasm to nuclei induced by irradiation and downregulates the transcription and expression of iNOS. Consequently, a combination of β-Elemene and irradiation treatment may promote radio-sensitization [67].

Lastly, a series of novel Palladium (II) complexes containing heterocyclic NO chelators appended to andrographolide were recently evaluated in vitro. These complexes demonstrated significant radical scavenging activity against a range of free radicals, including NO, outperforming standard antioxidants. Furthermore, they induced substantial cell death through apoptosis in the A549 human lung cancer cell line, exhibiting greater efficacy than the widely used chemotherapeutic drug cisplatin [68].

### 3.2. NO-Based Therapeutic Strategies

Another potential therapeutic strategy to impair tumor growth, decrease tumor angiogenesis, and control metastatic potential is to administer NO exogenously or to utilize a NO donor using drug delivery systems (DDS) to potentiate these mechanisms [69].

Preclinical studies utilizing ultra-high concentration gaseous NO (UHCgNO) have demonstrated its potential as a potent anti-neoplastic agent and its use as a novel method for tumor ablation. The anti-neoplastic effect may be mediated by reactive NO species generation, such as peroxynitrite, that can oxidize DNA and induce single-strand breaks. Additionally, UHCgNO induces apoptosis through the accumulation of the tumor suppressor protein p53, mitochondrial damage, alterations in the expression of members of the anti-apoptotic Bcl-2 family, caspase activation, and DNA fragmentation.

Tumor ablation mediated by UHCgNO offers several advantages. Its highly diffusible nature allows for good distribution within the tumor, though its short half-life limits its overall effect.

Additionally, short-term treatment can stimulate an immune-mediated anti-tumor response. It has been demonstrated that NO activates innate and adaptive immune system responses against tumors in a concentration-dependent manner, even when the primary tumor is not completely ablated. This suggests that it may be sufficient to destroy only part of the tumor to expose immune cells to cancer antigens.

The administration of UHCgNO to murine models bearing Lewis lung carcinoma cell lines demonstrated that T cells and dendritic cells were able to penetrate the tumor within 14 days. Additionally, there is an increase in T and B cells in both the spleen and blood, while there is a reduction in polymorphonuclear myeloid-derived suppressor cells (MDSCs) in the spleen 21 days following treatment. Tumor cells undergoing apoptosis release damage-associated molecular patterns (DAMPs), which recruit antigen-presenting cells (APCs) to process and present tumor antigens to T cells, thereby stimulating T cell proliferation and maturation and initiating a long-term immune response. These findings indicate that the UHCgNO ablation method may have therapeutic potential as an immunomodulating agent. 

Moreover, endogenous NO production in tumor adjacent blood vessels may limit cancer cells’ intravasation by causing DNA damage and apoptosis of malignant cells. Additionally, gNO may enhance the efficacy of chemotherapy and radiotherapy in resistant cancer cells by improving tumor blood flow and the delivery of drugs and oxygen [70].

There is increasing interest in developing NO-releasing compounds that can generate NO in particular tissues, avoiding systemic toxic effects. Exogenously supplied NO donors could induce cell death in human lung carcinoma.

This action can be explicated through a NO-mediated downregulation of survivin expression, a protein herein most tumor cells associated with both anti-apoptosis and mitotic progression [71]. NO donors S-nitroso-N-acetyl-penicillamine (SNAP) and sodium nitroprusside (SNP) decrease survivin expression via the p38 MAP kinase-dependent pathway, inducing apoptosis in lung carcinoma cells. SNAP inhibits cell growth and increases the G2/M fractions in lung cancer cells; the activation of the CDC2-cyclin B1 complex is required for the mitotic entry, and it was found that NO decreases the level of cyclin B1 and inhibits CDC2 kinase activity, causing the inhibition of survivin activity with enhanced NO-induced cell death. Moreover, NO may reduce radio- and chemoresistance through lowering survivin levels in tumor cells, considering survivin expression is enhanced during administration of several anti-cancer agents [72].

Another promising class of NO-based therapy is diazeniumdiolate-based NO-releasing prodrugs, such as O2-(2,4-dinitrophenyl)-1-[(4-ethoxycarbonyl) piperazin-1-yl] diazen-1-ium-1,2-diolate (JS-K). JS-K has a strong inhibitory effect on a subset of NSCLC cancer cell lines, and its effectiveness has a strong and statistically significant positive correlation with endogenous pre-existing levels of ROS/RNS, for which high levels are required for the generation of additional ROS/RNS. In mitochondria, NO determines increased superoxide generation and DNA damage by the inactivation of manganese superoxide dismutase through nitration. Beyond the alteration of redox balance in tumor cells, JS-K decreases the reduced levels of glutathione (GSH) and increases its oxidized form (GSSG). This leads to the activation of the intrinsic apoptotic pathway initiator Bax and consequent translocation to mitochondria, which triggers cytochrome c release from mitochondria [73].

Tumor lung metastasis is mediated by epithelial–mesenchymal transition (EMT) and endothelial–mesenchymal transition EMT (EndMT), promoted by TGF-β1 and epigenetic reprogramming, leading to a loss of typical epithelial (such as E-cadherin) or endothelial markers (such as CD31, CD34, VE-cadherin, and von Willebrand factor) and the acquisition of mesenchymal markers, such as vimentin and alpha smooth muscle actin (αSMA). The Zn-based metal nonoate [Zn (PipNONO)Cl] releases NO, serving as a regulator of EMT by interfering negatively with the expression and activity of TGF-β1, thus inhibiting cancer progression. Therefore, [Zn(PipNONO)Cl] may be a promising drug, acting on both tumor and endothelial cells, and reprogramming the cells toward their physiological phenotypes [69].

The effect of NO is influenced by a multitude of factors, including the kinetics of NO release by NO-based drugs, the availability of said drugs within the tumor microenvironment, and the presence of reactive oxygen species or other scavengers during the process of drug distribution [73].

**Figure 3:**
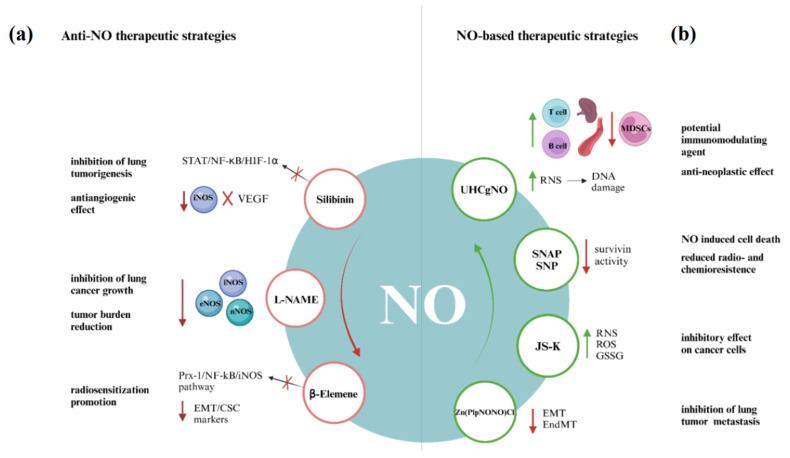
NO-centered therapeutic strategies in lung cancer: (**a**) Therapies inhibiting NO production by targeting the molecular pathways involved, such as the STAT/NF-κB/HIF-1α pathway, by silibinin. L-NAME acts as a pan-inhibitor of NOS enzymes, while β-Elemene inhibits the Prx-1/NF-kB/iNOS signaling pathway and reduces the expression of EMT/CSC markers. (**b**) Therapies based on exogenous administration of NO to potentiate its anti-tumor effects. UHCgNO increases the RNS levels, causing DNA damage and, from an immunological perspective, causes a rise in T and B cells in both the spleen and blood, while the MDSC levels decrease in the spleen. SNAP and SNP decrease survival activity, inducing cancer cell death. JS-K increases the RNS, ROS, and GSSG levels, determining an inhibitory effect on cancer cells. [Zn (PipNONO)Cl] inhibits EMT and EndMT by interfering negatively with the expression and activity of TGF-β1, inhibiting cancer progression.

All the presented therapeutic strategies, graphically summarized in Figure 3, were applied in the in vitro or in vivo mouse model studies. Indeed, for cancer therapy, the usefulness of aqueous solutions of NO and NO donors is highly restricted due to their brief half-lives, instability under physiological conditions, fast system clearance, non-specific NO release, and NO-independent toxicities. The advent of nanotechnology offers the potential for the development of a targeted therapeutic strategy that enables the regulated release of NO within the tumor microenvironment [74,75].

In light of the characteristics of the tumor microenvironment and the inherent instability of the NO molecule, NO donors that can be released in response to external factors have attracted considerable attention. In particular, it is possible to distinguish between donors that are sensitive to endogenous stimuli and donors that are sensitive to exogenous stimuli [75].

Endogenous stimuli include pH, glutathione (GSH), nitric oxide synthase 2 (NOS2), and glucose.

NONOates—widely used NO donors—are activated under acidic conditions such as those found in the tumor microenvironment. Their decomposition rate depends on both the solution pH and the substituents on the secondary amine groups. In contrast, high intracellular concentrations of GSH in cancer cells can trigger NO release from phenylsulfonyl furoxan (PSF) and nitrate-based polymers. Additionally, L-arginine, a natural precursor of NO, can function as a glucose-responsive NO donor, as glucose oxidase (GOx) catalyzes the conversion of glucose into gluconic acid and hydrogen peroxide (H_2_O_2_), the latter of which promotes NO release from L-arginine [75,76].

Conversely, external stimuli that are capable of inducing NO release include ultraviolet (UV) and near-infrared (NIR) light, in addition to X-rays. In view of the phototoxicity of UV rays, NIR-sensitive NO donors are the preferred option for achieving deeper tissue penetration. S-nitrosothiols (SNO) represent a class of NO donors that exhibit a multiple response, being activated by NIR, heat, and X-rays [75,77].

It has been reported that the use of biopolymers, such as polyethylene glycol gel, facilitates the delivery of NO to tumor cells that have been exposed to light, thereby inducing anti-tumor effects [78].

Another feature that can be exploited for the use of NO for anti-tumor purposes is hypoxia, which is known to be a prevalent feature of solid tumors, contributing to their progression through the stabilization of HIF-1α, a process that regulates genes involved in angiogenesis and cell survival. Indeed, it has been demonstrated that certain NO donors, including sodium nitroprusside, nitroglycerin, and isosorbide dinitrate, possess the capacity to reduce HIF-1α levels in a hypoxic environment. It has been demonstrated that this process results in a reduced hypoxic microenvironment, which may consequently enhance therapeutic efficacy [79].

In the field of nanomedicine, other agents employed as nano-carriers include thermosensitive liposomes. These liposomes possess the capacity to store, transfer, and activate the release of NO in a heat-mediated manner [80,81]. Moreover, NO donors can be combined with fluorescent nanoparticles and can be used for photodynamic therapy of tumors [82,83].

An alternative strategy is the combination of a NO donor with other drugs, with the objective of maintaining the pharmacological activity of the parent drug while exploiting the biological actions of NO [84]. Combination strategies that have shown efficacy against NSCLC cell lines include the use of nitric oxide donors together with cisplatin and doxorubicin [85,86]. In parallel, recent advancements in nanotechnology have led to the development of innovative nanosystems that combine NO with chemotherapeutic agents. One such system, (ZnO, NONO)@Ves-PTX, was engineered to release NO under the acidic conditions characteristic of the tumor microenvironment (TME). The NO generated within the TME inhibits angiogenesis, thereby enhancing the delivery and distribution of therapeutic agents. Upon internalization by tumor cells, (ZnO, NONO)@Ves-PTX decomposes in response to intracellular GSH, releasing bilayer-encapsulated paclitaxel (PTX), which exerts its chemotherapeutic effects against lung cancer cells [87].

Nitric oxide-driven nanotherapeutics have also been used in combination with RT for the treatment of lung cancer. Resistance to RT in some lung cancer patients is often attributed to reduced DNA damage, cytoprotective mechanisms, and an impaired immune response following treatment. To address these challenges, a combined approach utilizing radiotherapy and S-nitrosated human serum albumin nanoparticles has been investigated. This combination has demonstrated a significant suppression of lung tumor growth, highlighting the potential of NO donors to enhance the efficacy of radiotherapy by sensitizing tumor cells and mitigating cytoprotective and immune-resistant effects [88].

Other therapeutic strategies developed over recent decades aim to provide a targeted delivery of NO at the tumor site. These include conjugating NO with antibodies to specifically target tumor cells [89] and employing gene therapy, which involves transferring a cDNA sequence encoding NOS into tumor cells [27,90].

It is anticipated that these therapeutic strategies will be evaluated in human models in the future, with the goal of developing targeted approaches for the treatment of lung cancer that minimize systemic side effects.

It is evident that NO exerts a dual effect on tumors, demonstrating both anti-tumor and carcinogenic properties. The manifestation of these effects is predominantly contingent on the concentration of NO [27]. This duality presents a double challenge in determining the net impact of NO on cancer and in defining the therapeutic role of NO-focused anti-tumor strategies. Currently, there is no clear evidence or guidelines defining a minimum or maximum cut-off value of the NO required to exert an anti-tumor effect. The instability of the NO molecule in the tumor microenvironment further complicates the definition of this value. Preclinical studies emphasize the importance of controlled and tumor-specific NO release strategies [14]. Nevertheless, a more profound and dynamic understanding of the influence of NO on the molecular and cellular mechanisms of tumor biology may enable researchers to fully exploit the anti-tumor potential of NO-targeting therapies.

However, further research employing standardized dose–response models and real-time monitoring of NO levels in the tumor microenvironment will be essential to establish clinically relevant threshold values.

## 4. Future Directions and Conclusion Remarks

Over the past decade, a better understanding of the complex pathogenesis of lung cancer has led to the development of new therapeutic options. However, despite advances in treatment, the overall prognosis

FeNO may help to assess the complications that are associated with multimodal treatments involving surgery and RT, including postoperative pneumonia and radiation pneumonitis. Several interesting issues remain to be solved regarding the impact of confounding and selection bias alongside the standardization of a reliable cut-off value. Further studies with a larger sample size are needed to confirm and validate the clinical utility of FeNO in predicting potentially preventable complications.

Due to the marked inhibitory effect of CHT on FeNO levels, the interpretation of FeNO kinetics during pharmacological treatment is still a largely unexplored issue. In recent years, the rise of biological treatment targeting different cancer pathways and/or enhancing the immune system’s responses may lead to a better evaluation of FeNO as a prognostic biomarker of treatment response.

The extensive role of NO in cancer development and progression has cast a spotlight on the therapeutic role of NO-targeting, while the bimodal effect of NO on carcinogenesis and cellular homeostasis has impeded its clinical use.

NO donors’ therapeutic strategies have been shown to increase the availability of NO, resulting in a chemotherapeutic and radiotherapeutic sensitizing activity. Nevertheless, the administration of exogenous NO, other than promoting the immunogenicity of cancer cells, also has immunosuppressive effects. This may result in accelerated tumor progression, as current NO delivery systems are limited in their ability to deliver high concentrations of NO only to the tumor microenvironment and not to other sites. Moreover, high systemic doses of NO may cause a reduction in arterial blood pressure due to the vasodilatory effects of NO. On the other hand, low concentrations of NO may be insufficient to exert anti-tumor action.

Moreover, due to NO chemical and biological features, such as its low half-life and rapid systemic clearance, its clinical efficacy in real-life settings is debated, and its toxicity profile is considerable.

The improvements of DDS, including liposomes and nanoparticles, may improve tumor targeting, constitute a major challenge of research, and may pave the way for more effective anti-cancer applications of NO.

## Figures and Tables

**Table 1:** Description of the main characteristics of NOS Isoforms.

Isoform	Descriptive Name	Molecular Weight (kD)	Gene Encoding and Position	Tissue	Expression	Subcellular Localization
NOS-1	neuronal NOS (nNOS)	155	NOS1 (12q24.2-31)	Neurons, skeletal muscle	Constitutive	Cytosol, endoplasmic reticulum, sarcolemma, postsynaptic densities, caveolae (caveolin 3)
NOS-2	inducible NOS (iNOS)	125	NOS2 (17q11.2-12)	Macrophages, smooth muscle cells	Transcriptional induction	Phagosomes
NOS-3	endothelial NOS (eNOS)	135	NOS3 (7q35-36)	Endothelial cells, neurons	Constitutive	Golgi apparatus, plasmalemmal caveolae

## Data Availability

No new data were created or analyzed in this study. Data sharing is not applicable to this article.

## Abbreviations

The following abbreviations are used in this manuscript:

APC	Antigen-presenting cell
CHT	Chemotherapy
DAMP	Damage-associated molecular patterns
DDS	Drug delivery system
EMT	Epithelial–mesenchymal transition
FeNO	Fractional exhaled nitric oxide
GSH	Glutathione
GSSG	Glutathione disulfide
L-NAME	NG-nitro-L-arginine methyl ester
MDSCs	Myeloid-derived suppressor cells
NO	Nitric oxide
NOS	Nitric oxide synthase
NSCLC	Non small cell lung cancer
POP	Postoperative pneumonia
PTX	Paclitaxel
ROS	Reactive oxygen species
RP	Radiation pneumonitis
RT	Radiotherapy
SNAP	S-nitroso-N-acetyl-penicillamine
SNP	Sodium nitroprusside
TME	Tumor microenvironment
UHCgNO	Ultra-high concentration gaseous NO

## References

[1] Andrabi S.M., Sharma N.S., Karan A., Shahriar S.M.S., Cordon B., Ma B., Xie J. (2023). Nitric Oxide: Physiological Functions, Delivery, and Biomedical Applications. Adv. Sci..

[2] Cinelli M.A., Do H.T., Miley G.P., Silverman R.B. (2020). Inducible Nitric Oxide Synthase: Regulation, Structure, and Inhibition. Med. Res. Rev..

[3] Fukumura D., Kashiwagi S., Jain R.K. (2006). The Role of Nitric Oxide in Tumour Progression. Nat. Rev. Cancer.

[4] Lala P.K., Orucevic A. (1998). Role of Nitric Oxide in Tumor Progression: Lessons from Experimental Tumors. Cancer Metastasis Rev..

[5] Burke A.J., Sullivan F.J., Giles F.J., Glynn S.A. (2013). The Yin and Yang of Nitric Oxide in Cancer Progression. Carcinogenesis.

[6] Ridnour L.A., Thomas D.D., Donzelli S., Espey M.G., Roberts D.D., Wink D.A., Isenberg J.S. (2006). The Biphasic Nature of Nitric Oxide Responses in Tumor Biology. Antioxid. Redox Signal..

[7] Perera F.P. (1997). Environment and Cancer: Who Are Susceptible?. Science.

[8] Yokota J., Shiraishi K., Kohno T. (2010). Genetic Basis for Susceptibility to Lung Cancer. Advances in Cancer Research.

[9] Grimm E.A., Sikora A.G., Ekmekcioglu S. (2013). Molecular Pathways: Inflammation-Associated Nitric-Oxide Production as a Cancer-Supporting Redox Mechanism and a Potential Therapeutic Target. Clin. Cancer Res..

[10] Warren G.W., Cummings K.M. (2013). Tobacco and Lung Cancer: Risks, Trends, and Outcomes in Patients with Cancer. Am. Soc. Clin. Oncol. Educ. Book.

[11] Pryor W.A., Stone K. (1993). Oxidants in Cigarette Smoke Radicals, Hydrogen Peroxide, Peroxynitrate, and Peroxynitritea. Ann. N. Y. Acad. Sci..

[12] Zi Y., Wang X., Zi Y., Yu H., Lan Y., Fan Y., Ren C., Liao K., Chen H. (2023). Cigarette Smoke Induces the ROS Accumulation and iNOS Activation through Deactivation of Nrf-2/SIRT3 Axis to Mediate the Human Bronchial Epithelium Ferroptosis. Free Radic. Biol. Med..

[13] Salimian Rizi B., Achreja A., Nagrath D. (2017). Nitric Oxide: The Forgotten Child of Tumor Metabolism. Trends Cancer.

[14] Alimoradi H., Greish K., Gamble A.B., Giles G.I. (2019). Controlled Delivery of Nitric Oxide for Cancer Therapy. Pharm. Nanotechnol..

[15] Bellary A., Nowak C., Iwanicki I., Flores-Guzman F., Wu L., Kandel J.J., Laetsch T.W., Bleris L., Hernandez S.L., Sirsi S.R. (2023). Non-Viral Nitric Oxide-Based Gene Therapy Improves Perfusion and Liposomal Doxorubicin Sonopermeation in Neuroblastoma Models. Theranostics.

[16] Iwasaki T., Higashiyama M., Kuriyama K., Sasaki A., Mukai M., Shinkai K., Horai T., Matsuda H., Akedo H. (1997). N^G^-Nitro-L-arginine Methyl Ester Inhibits Bone Metastasis after Modified Intracardiac Injection of Human Breast Cancer Cells in a Nude Mouse Model. Jpn. J. Cancer Res..

[17] Bonavida B., Baritaki S., Huerta-Yepez S., Vega M.I., Jazirehi A.R., Berenson J., Bonavida B. (2010). Nitric Oxide Donors Are a New Class of Anti-Cancer Therapeutics for the Reversal of Resistance and Inhibition of Metastasis. Nitric Oxide (NO) and Cancer.

[18] Mocellin S., Bronte V., Nitti D. (2007). Nitric Oxide, a Double Edged Sword in Cancer Biology: Searching for Therapeutic Opportunities. Med. Res. Rev..

[19] Menzies-Gow A., Mansur A.H., Brightling C.E. (2020). Clinical Utility of Fractional Exhaled Nitric Oxide in Severe Asthma Management. Eur. Respir. J..

[20] Pianigiani T., Alderighi L., Meocci M., Messina M., Perea B., Luzzi S., Bergantini L., D’Alessandro M., Refini R.M., Bargagli E. (2023). Exploring the Interaction between Fractional Exhaled Nitric Oxide and Biologic Treatment in Severe Asthma: A Systematic Review. Antioxidants.

[21] Cameli P., Bargagli E., Bergantini L., d’Alessandro M., Pieroni M., Fontana G.A., Sestini P., Refini R.M. (2020). Extended Exhaled Nitric Oxide Analysis in Interstitial Lung Diseases: A Systematic Review. Int. J. Mol. Sci..

[22] Liu X., Zhang H., Wang Y., Lu Y., Gao Y., Lu Y., Zheng C., Yin D., Wang S., Huang K. (2020). Fractional Exhaled Nitric Oxide Is Associated with the Severity of Stable COPD. COPD J. Chronic Obstr. Pulm. Dis..

[23] Miranda K.M., Ridnour L.A., McGinity C.L., Bhattacharyya D., Wink D.A. (2021). Nitric Oxide and Cancer: When to Give and When to Take Away?. Inorg. Chem..

[24] Mintz J., Vedenko A., Rosete O., Shah K., Goldstein G., Hare J.M., Ramasamy R., Arora H. (2021). Current Advances of Nitric Oxide in Cancer and Anticancer Therapeutics. Vaccines.

[25] Khan F.H., Dervan E., Bhattacharyya D.D., McAuliffe J.D., Miranda K.M., Glynn S.A. (2020). The Role of Nitric Oxide in Cancer: Master Regulator or NOt?. Int. J. Mol. Sci..

[26] Smolarz B., Łukasiewicz H., Samulak D., Piekarska E., Kołaciński R., Romanowicz H. (2025). Lung Cancer—Epidemiology, Pathogenesis, Treatment and Molecular Aspect (Review of Literature). Int. J. Mol. Sci..

[27] Hu Y., Xiang J., Su L., Tang X. (2020). The Regulation of Nitric Oxide in Tumor Progression and Therapy. J. Int. Med. Res..

[28] Klebe S., Leigh J., Henderson D.W., Nurminen M. (2019). Asbestos, Smoking and Lung Cancer: An Update. Int. J. Environ. Res. Public. Health.

[29] Sinha B.K. (2023). Can Nitric Oxide-Based Therapy Be Improved for the Treatment of Cancers? A Perspective. Int. J. Mol. Sci..

[30] Bonavida B. (2020). Sensitizing Activities of Nitric Oxide Donors for Cancer Resistance to Anticancer Therapeutic Drugs. Biochem. Pharmacol..

[31] Li Y., Yoon B., Dey A., Nguyen V.Q., Park J.H. (2022). Recent Progress in Nitric Oxide-Generating Nanomedicine for Cancer Therapy. J. Control. Release.

[32] Zhou H., Li J., Chen Z., Chen Y., Ye S. (2021). Nitric Oxide in Occurrence, Progress and Therapy of Lung Cancer: A Systemic Review and Meta-Analysis. BMC Cancer.

[33] Cao H., Chen X., Song Y., Xue T., Xue Z., Zhang G., Wang K., Liu Z. (2024). Comparing the Utility of Lung Function Parameters and Fractional Exhaled Nitric Oxide in Predicting Lung Cancer. Sarcoidosis Vasc. Diffus. Lung Dis..

[34] Okamoto K., Hayashi K., Kaku R., Kawaguchi Y., Oshio Y., Hanaoka J. (2020). Impact of Fractional Exhaled Nitric Oxide on the Outcomes of Lung Resection Surgery: A Prospective Study. J. Thorac. Dis..

[35] Liu G.-X., Yang Y., Chen L., Gu M.-Q., He J.-T., Wang X. (2022). Perioperative Exhaled Nitric Oxide as an Indicator for Postoperative Pneumonia in Surgical Lung Cancer Patients: A Prospective Cohort Study Based on 183 Cases. Can. Respir. J..

[36] Lin R., Yu G., Tu X. (2024). Preoperative Fractional Exhaled Nitric Oxide Is a Risk and Predictive Factor of Postoperative Cough for Early-Stage Non-Small Cell Lung Cancer Patients: A Longitudinal Study. BMC Pulm. Med..

[37] Käsmann L., Dietrich A., Staab-Weijnitz C.A., Manapov F., Behr J., Rimner A., Jeremic B., Senan S., De Ruysscher D., Lauber K. (2020). Radiation-Induced Lung Toxicity—Cellular and Molecular Mechanisms of Pathogenesis, Management, and Literature Review. Radiat. Oncol. Lond. Engl..

[38] Voruganti Maddali I.S., Cunningham C., McLeod L., Bahig H., Chaudhuri N., Chua K.L.M., Evison M., Faivre-Finn C., Franks K., Harden S. (2024). Optimal Management of Radiation Pneumonitis: Findings of an International Delphi Consensus Study. Lung Cancer.

[39] Shepherd A.F., Iocolano M., Leeman J., Imber B.S., Wild A.T., Offin M., Chaft J.E., Huang J., Rimner A., Wu A.J. (2021). Clinical and Dosimetric Predictors of Radiation Pneumonitis in Patients with Non-Small Cell Lung Cancer Undergoing Postoperative Radiation Therapy. Pract. Radiat. Oncol..

[40] Moré J.M., Eclov N.C.W., Chung M.P., Wynne J.F., Shorter J.H., Nelson D.D., Hanlon A.L., Burmeister R., Banos P., Maxim P.G. (2014). Feasibility and Potential Utility of Multicomponent Exhaled Breath Analysis for Predicting Development of Radiation Pneumonitis After Stereotactic Ablative Radiotherapy. J. Thorac. Oncol..

[41] McCurdy M.R., Wazni M.W., Martinez J., McAleer M.F., Guerrero T. (2011). Exhaled Nitric Oxide Predicts Radiation Pneumonitis in Esophageal and Lung Cancer Patients Receiving Thoracic Radiation. Radiother. Oncol..

[42] Enache I., Noel G., Jeung M.-Y., Meyer N., Oswald-Mammosser M., Urban-Kraemer E., Schumacher C., Geny B., Quoix E., Charloux A. (2012). Can Exhaled NO Fraction Predict Radiotherapy-Induced Lung Toxicity in Lung Cancer Patients?. Radiat. Oncol..

[43] Szejniuk W.M., Nielsen M.S., Brønnum D., Takács-Szabó Z., Weinreich U.M., Pilegaard Thomsen L., Bøgsted M., Jensen I., McCulloch T., Falkmer U.G. (2019). Fractional Exhaled Nitric Oxide as a Potential Biomarker for Radiation Pneumonitis in Patients with Non-Small Cell Lung Cancer: A Pilot Study. Clin. Transl. Radiat. Oncol..

[44] Rittmeyer A., Barlesi F., Waterkamp D., Park K., Ciardiello F., Von Pawel J., Gadgeel S.M., Hida T., Kowalski D.M., Dols M.C. (2017). Atezolizumab versus Docetaxel in Patients with Previously Treated Non-Small-Cell Lung Cancer (OAK): A Phase 3, Open-Label, Multicentre Randomised Controlled Trial. Lancet.

[45] Fehrenbacher L., Spira A., Ballinger M., Kowanetz M., Vansteenkiste J., Mazieres J., Park K., Smith D., Artal-Cortes A., Lewanski C. (2016). Atezolizumab versus Docetaxel for Patients with Previously Treated Non-Small-Cell Lung Cancer (POPLAR): A Multicentre, Open-Label, Phase 2 Randomised Controlled Trial. Lancet.

[46] Mirzaei S., Hushmandi K., Zabolian A., Saleki H., Torabi S.M.R., Ranjbar A., SeyedSaleh S., Sharifzadeh S.O., Khan H., Ashrafizadeh M. (2021). Elucidating Role of Reactive Oxygen Species (ROS) in Cisplatin Chemotherapy: A Focus on Molecular Pathways and Possible Therapeutic Strategies. Molecules.

[47] Srivastava A.N., Gupta A., Srivastava S., Natu S.M., Mittal B., Negi M.P.S., Prasad R. (2010). Cisplatin Combination Chemotherapy Induces Oxidative Stress in Advance Non Small Cell Lung Cancer Patients. Asian Pac. J. Cancer Prev. APJCP.

[48] Wewel A.R., Crusius J.A.M., Gatzemeier U., Heckmayr M., Becher G., Magnussen H., Jörres R.A., Holz O. (2006). Time Course of Exhaled Hydrogen Peroxide and Nitric Oxide during Chemotherapy. Eur. Respir. J..

[49] Wu C., Liu W., Pu J., Feng T., Chang Y., Wang X., Liang X., Kai J. (2022). Fractional exhaled nitric oxide in checkpoint inhibitor pneumonitis: A case report and literature review. Immunotherapy.

[50] Suzuki Y., Saito J., Kubota S., Ikeda M., Rikimaru M., Yamada R., Kumanaka T., Tanaka R., Kazama K., Saito K. (2025). Successful management of chronic eosinophilic pneumonia triggered by immune checkpoint inhibitor: A case report and literature review. Front Immunol..

[51] Hayakawa Y., Kawaguchi T., Yamasaki K., Endo M., Komatsu M., Ishiguro Y., Murata Y., Yatera K. (2023). Immune checkpoint inhibitor-induced asthma and chronic obstructive pulmonary disease overlap in patient with adenocarcinoma. Respirol Case Rep..

[52] Watanabe H., Asada K., Shirai T., Torii H., Yoshimura K., Kusafuka K. (2020). Eosinophilic airway inflammation and eosinophilic chronic rhinosinusitis during nivolumab and ipilimumab. Respirol Case Rep..

[53] Gao Y., Luo T., Huang D., Fu Z., Ma S., Lin L., Huang H., Liu T., Zhang J., Jiang X. (2025). Construction of a checkpoint inhibitor-related pneumonia diagnostic model based on exhaled nitric oxide: A prospective observational study. Transl Lung Cancer Res..

[54] Agustí A., Bafadhel M., Beasley R., Bel E.H., Faner R., Gibson P.G., Louis R., McDonald V.M., Sterk P.J., Thomas M. (2017). Precision Medicine in Airway Diseases: Moving to Clinical Practice. Eur. Respir. J..

[55] Donohue J.F., Jain N. (2013). Exhaled Nitric Oxide to Predict Corticosteroid Responsiveness and Reduce Asthma Exacerbation Rates. Respir. Med..

[56] Ragnoli B., Radaeli A., Pochetti P., Kette S., Morjaria J., Malerba M. (2023). Fractional Nitric Oxide Measurement in Exhaled Air (FeNO): Perspectives in the Management of Respiratory Diseases. Ther. Adv. Chronic Dis..

[57] Eckel S.P., Linn W.S., Berhane K., Rappaport E.B., Salam M.T., Zhang Y., Gilliland F.D. (2014). Estimation of Parameters in the Two-Compartment Model for Exhaled Nitric Oxide. PLoS ONE.

[58] Lehtimäki L., Turjanmaa V., Kankaanranta H., Saarelainen S., Hahtola P., Moilanen E. (2000). Increased Bronchial Nitric Oxide Production in Patients with Asthma Measured with a Novel Method of Different Exhalation Flow Rates. Ann. Med..

[59] Paraskakis E., Vergadi E., Chatzimichael A., Bush A. (2016). The Role of Flow-Independent Exhaled Nitric Oxide Parameters in the Assessment of Airway Diseases. Curr. Top. Med. Chem..

[60] Okamoto K., Kawaguchi Y., Shiratori T., Oshio Y., Hanaoka J. (2024). Clinicopathological Study of Fractional Exhaled Nitric Oxide Dynamics and Intratumoral Inducible Nitric Oxide Synthase Expression in Primary Lung Cancer Patients. Transl. Cancer Res..

[61] Verdura S., Cuyàs E., Ruiz-Torres V., Micol V., Joven J., Bosch-Barrera J., Menendez J.A. (2021). Lung Cancer Management with Silibinin: A Historical and Translational Perspective. Pharmaceuticals.

[62] Ramasamy K., Dwyer-Nield L.D., Serkova N.J., Hasebroock K.M., Tyagi A., Raina K., Singh R.P., Malkinson A.M., Agarwal R. (2011). Silibinin Prevents Lung Tumorigenesis in Wild-Type but Not in iNOS^−/−^ Mice: Potential of Real-Time Micro-CT in Lung Cancer Chemoprevention Studies. Clin. Cancer Res..

[63] Chittezhath M., Deep G., Singh R.P., Agarwal C., Agarwal R. (2008). Silibinin Inhibits Cytokine-Induced Signaling Cascades and down-Regulates Inducible Nitric Oxide Synthase in Human Lung Carcinoma A549 Cells. Mol. Cancer Ther..

[64] Pershing N.L.K., Yang C.-F.J., Xu M., Counter C.M. (2016). Treatment with the Nitric Oxide Synthase Inhibitor L-NAME Provides a Survival Advantage in a Mouse Model of *Kras.* Mutation-Positive, Non-Small Cell Lung Cancer. Oncotarget.

[65] Javrushyan H., Ginovyan M., Harutyunyan T., Gevorgyan S., Karabekian Z., Maloyan A., Avtandilyan N. (2025). Elucidating the Impact of Hypericum Alpestre Extract and L-NAME on the PI3K/Akt Signaling Pathway in A549 Lung Adenocarcinoma and MDA-MB-231 Triple-Negative Breast Cancer Cells. PLoS ONE.

[66] Bai Z., Yao C., Zhu J., Xie Y., Ye X.-Y., Bai R., Xie T. (2021). Anti-Tumor Drug Discovery Based on Natural Product β-Elemene: Anti-Tumor Mechanisms and Structural Modification. Molecules.

[67] Zou K., Li Z., Zhang Y., Mu L., Chen M., Wang R., Deng W., Zou L., Liu J. (2021). β-Elemene Enhances Radiosensitivity in Non-Small-Cell Lung Cancer by Inhibiting Epithelial–Mesenchymal Transition and Cancer Stem Cell Traits via Prx-1/NF-kB/iNOS Signaling Pathway. Aging.

[68] Prasad P., Parveen S., Alarfaj A.A., Hirad A.H., Subarkhan M.M., Dhanapal S., Kalaiarasi G. (2025). Palladium(II) Complexes Containing Andrographolide Appended N,O Heterocyclic Chelators: Investigation of Anti-Oxidant, Anti-Cancer and Apoptotic Activities. J. Inorg. Biochem..

[69] Ciccone V., Filippelli A., Bacchella C., Monzani E., Morbidelli L. (2022). The Nitric Oxide Donor [Zn(PipNONO)Cl] Exhibits Antitumor Activity through Inhibition of Epithelial and Endothelial Mesenchymal Transitions. Cancers.

[70] Confino H., Dirbas F.M., Goldshtein M., Yarkoni S., Kalaora R., Hatan M., Puyesky S., Levi Y., Malka L., Johnson M. (2022). Gaseous Nitric Oxide Tumor Ablation Induces an Anti-Tumor Abscopal Effect. Cancer Cell Int..

[71] Pachimatla A.G., Fenstermaker R., Ciesielski M., Yendamuri S. (2024). Survivin in Lung Cancer: A Potential Target for Therapy and Prevention—A Narrative Review. Transl. Lung Cancer Res..

[72] Chao J.-I., Kuo P.-C., Hsu T.-S. (2004). Down-Regulation of Survivin in Nitric Oxide-Induced Cell Growth Inhibition and Apoptosis of the Human Lung Carcinoma Cells. J. Biol. Chem..

[73] Maciag A.E., Chakrapani H., Saavedra J.E., Morris N.L., Holland R.J., Kosak K.M., Shami P.J., Anderson L.M., Keefer L.K. (2011). The Nitric Oxide Prodrug JS-K Is Effective against Non–Small-Cell Lung Cancer Cells In Vitro and In Vivo: Involvement of Reactive Oxygen Species. J. Pharmacol. Exp. Ther..

[74] Zhao Z., Shan X., Zhang H., Shi X., Huang P., Sun J., He Z., Luo C., Zhang S. (2023). Nitric Oxide-Driven Nanotherapeutics for Cancer Treatment. J. Control. Release.

[75] Gao D., Asghar S., Hu R., Chen S., Niu R., Liu J., Chen Z., Xiao Y. (2023). Recent Advances in Diverse Nanosystems for Nitric Oxide Delivery in Cancer Therapy. Acta Pharm. Sin. B.

[76] Hrabie J.A., Keefer L.K. (2002). Chemistry of the Nitric Oxide-Releasing Diazeniumdiolate (“Nitrosohydroxylamine”) Functional Group and Its Oxygen-Substituted Derivatives. Chem. Rev..

[77] Fan W., Lu N., Huang P., Liu Y., Yang Z., Wang S., Yu G., Liu Y., Hu J., He Q. (2017). Glucose-Responsive Sequential Generation of Hydrogen Peroxide and Nitric Oxide for Synergistic Cancer Starving-Like/Gas Therapy. Angew. Chem..

[78] Wang Z., Ye Q., Yu S., Akhavan B. (2023). Poly Ethylene Glycol (PEG)-Based Hydrogels for Drug Delivery in Cancer Therapy: A Comprehensive Review. Adv. Healthc. Mater..

[79] Bonfili L., Gong C., Lombardi F., Cifone M.G., Eleuteri A.M. (2021). Strategic Modification of Gut Microbiota through Oral Bacteriotherapy Influences Hypoxia Inducible Factor-1α: Therapeutic Implication in Alzheimer’s Disease. Int. J. Mol. Sci..

[80] Haemmerich D., Ramajayam K.K., Newton D.A. (2023). Review of the Delivery Kinetics of Thermosensitive Liposomes. Cancers.

[81] Tai L.-A., Wang Y.-C., Yang C.-S. (2010). Heat-Activated Sustaining Nitric Oxide Release from Zwitterionic Diazeniumdiolate Loaded in Thermo-Sensitive Liposomes. Nitric Oxide.

[82] Lin Z., Zhu T., Zhong X. (2024). NIR-Triggered NO Production Combined with Photodynamic Therapy for Tumor Treatment. Photodiagn. Photodyn. Ther..

[83] Girotti W.A., Fahey M.J., Korytowski W. (2016). Multiple Means by Which Nitric Oxide Can Antagonize Photodynamic Therapy. Curr. Med. Chem..

[84] Li C.-Y., Anuraga G., Chang C.-P., Weng T.-Y., Hsu H.-P., Ta H.D.K., Su P.-F., Chiu P.-H., Yang S.-J., Chen F.-W. (2023). Repurposing Nitric Oxide Donating Drugs in Cancer Therapy through Immune Modulation. J. Exp. Clin. Cancer Res..

[85] Munaweera I., Shi Y., Koneru B., Patel A., Dang M.H., Di Pasqua A.J., Balkus K.J. (2015). Nitric Oxide- and Cisplatin-Releasing Silica Nanoparticles for Use against Non-Small Cell Lung Cancer. J. Inorg. Biochem..

[86] Romano B., Molaro M.C., Somma F., Battisegola C., Failla M., Lazzarato L., Chegaev K., Rolando B., Kopecka J., Ianaro A. (2025). FS536, a Novel Nitric Oxide-Releasing Doxorubicin Hybrid, Reverts Multidrug Resistance in Lung Cancer Cells. J. Control. Release.

[87] Wang D., Qiu C.-J., Chu Y., Zhang A., Huang R., Pan S.-J., Tan L. (2024). A Polymeric Vesicle System for Combined Lung Cancer Therapy through Chemotherapy and Vasculature Normalization. Biomater. Res..

[88] Hu R., Jiang X., Zhu L., Meng R., Yang R., Sun W., Zhao Z., Lyu Y., Huang R., Xue F. (2025). Overcoming Radiation-Induced PD-L1 and COX-2 Upregulation by Nitric Oxide Gas Nanogenerator to Sensitize Radiotherapy of Lung Cancer. Biomaterials.

[89] Sun F., Wang Y., Luo X., Ma Z., Xu Y., Zhang X., Lv T., Zhang Y., Wang M., Huang Z. (2019). Anti-CD24 Antibody–Nitric Oxide Conjugate Selectively and Potently Suppresses Hepatic Carcinoma. Cancer Res..

[90] Bolli R. (2001). Cardioprotective Function of Inducible Nitric Oxide Synthase and Role of Nitric Oxide in Myocardial Ischemia and Preconditioning: An Overview of a Decade of Research. J. Mol. Cell. Cardiol..

